# MicroRNA-129-5p inhibits 3T3-L1 preadipocyte proliferation by targeting G3BP1

**DOI:** 10.1080/19768354.2017.1337046

**Published:** 2017-06-15

**Authors:** Shun Lv, Meilin Ma, Yunmei Sun, Xiangming Wang, Naren Qimuge, Jin Qin, Weijun Pang

**Affiliations:** Laboratory of Animal Fat Deposition and Muscle Development, College of Animal Science and Technology, Northwest A&F University, Yangling, Shaanxi Province, People’s Republic of China

**Keywords:** MiR-129-5p, 3T3-L1 preadipocyte, cell proliferation, G3BP1, p38 signaling pathway

## Abstract

MicroRNAs have been regarded to play a crucial role in the proliferation of different cell types including preadipocytes. In our study, we observed that miR-129-5p was down-regulated during 3T3-L1 preadipocyte proliferation, while the expression of G3BP1 showed a contrary tendency. 5-Ethynyl-2′-deoxyuridine (EdU) incorporation assay and flow cytometry showed that overexpression of miR-129-5p could bring about a reduction in S-phase cells and G2-phase arrest. Additional study indicated that miR-129-5p impaired cell cycle-related genes in 3T3-L1 preadipocytes. Importantly, it showed that miR-129-5p directly targeted the 3′UTR of G3BP1 and the expression of G3BP1 was inhibited by miR-129-5p mimic. Moreover, miR-129-5p mimic activated the p38 signaling pathway through up-regulating p38 and the phosphorylation level of p38. In a word, results in our study revealed that miR-129-5p suppressed preadipocyte proliferation via targeting G3BP1 and activating the p38 signaling pathway.

## Introduction

MicroRNAs are a class of endogenous 19–23 nt RNAs that always play an important part in the regulation of gene function by pairing with mRNAs and guide the posttranscriptional repression of coding genes (Bartel 2009). Lin-4 was discovered as the first miRNA (Lee et al. 1993); since then, large quantities of miRNAs have been identified and researches have shown that most miRNAs are evolutionarily conserved (Kapranov et al. 2002). Lots of studies have demonstrated that microRNAs could regulate many biological processes in a posttranscriptional way (Hobert 2008), including differentiation (Kim et al. 2006), proliferation (Brennecke et al. 2003) and apoptosis (Cheng et al. 2005). However, there are many miRNAs unexplored and further studies are required to gain a comprehensive view on how miRNA regulates biological processes.

Because the increase in white adipose tissue (WAT) mass in obese people increases the risk of type 2 diabetes mellitus (Yach et al. 2006), obesity is a serious threat to health worldwide. The increase in preadipocyte proliferation and subsequent differentiation into mature adipocytes result in an increase in the number of adipocytes (Spiegelman & Flier 1996). Present studies have shown that many regulatory factors are involved in the preadipocyte proliferation, including microRNAs. Skp2 regulated the proliferation of preadipocytes in the obesity development process (Sakai et al. 2007). The inhibition of miR-26b could promote human preadipocyte proliferation (Song et al. 2014), while miR-139-5p was shown to impair the clonal expansion of 3T3-L1 cells (Mi et al. 2015). But the molecular mechanism involved in preadipocyte proliferation still needs further discovery.

MicroRNA-129 was regarded to play a potential regulator role in tumor. miR-129-3p and miR-129-5p are mature miRNAs, which are processed from 3′ and 5′ precursors of miR-129, respectively (Lagos-Quintana et al. 2002). MiR-129-3p could regulate CP110 to impair cilia assembly; moreover, it was also found to play an important role in colorectal cancer (Cao et al. 2012; Liu et al. 2012b). MiR-129-5p has been proved to be involved in the control of many cancer processes. MiR-129-5p targeted CDK6 and caused G1-phase arrest to suppress cell proliferation in lung tumor cells (Wu et al. 2010). Also, miR-129-5p decreased VCP/p97 to suppress the development of liver cancer (Liu et al. 2012a). A miRNA microarray analysis showed that miR-129-5p was highly expressed in insulin-resistant 3T3-L1 preadipocytes (Ling et al. 2009), which shows a probable role of miR-129-5p in obesity. G3BP1, GTPase-activating protein SH3 domain-binding protein 1, could bind to Ras-GAP SH3 domain (Parker et al. 1996). G3BP1 was also known to facilitate cell proliferation by inhibiting PMP22 in breast cancer cells (Winslow et al. 2013). However, although many researches have demonstrated a tumor suppression role of miR-129-5p, its function in preadipocyte proliferation was not clear.

Here, we overexpressed miR-129-5p in 3T3-L1 preadipocytes and found that it could regulate not only DNA synthesis, but also the expression of proliferation marker genes. Further researches suggested that miR-129-5p inhibited preadipocyte proliferation by targeting G3BP1 and activating the p38 signaling pathway.

## Materials and methods

### Animals

C57BL mice were purchased from the Fourth Military Medical University Animal Center (Xi’an, China) and preserved in Laboratory of Animal Fat Deposition and Muscle Development. Nine tissues including liver, heart, spleen, kidney, brain, skeletal muscle, visceral adipose tissue (VAT), subcutaneous adipose tissue (SAT) and brown adipose tissue (BAT) were harvested from 10-week-old mice. These tissues were collected and analyzed following the requirements of Northwest Agriculture and Forest University Ethics Committee.

### Cell culture and transfection

3T3-L1 cells and HEK293T cells were incubated at 37°C and 5% CO_2_. The culture medium consists of Dulbecco’s modified Eagle’s medium (DMEM) and 10% fetal bovine serum (FBS). The medium was changed every 2 days. miR-129-5p mimic or negative control (NC) (RiboBio, Guangzhou, China) was transfected into 3T3 cells at 40% density using Roche Transfection Reagent following the specifications. We harvested cells at 24 and 48 h during cell proliferation for quantitative real-time PCR (qRT-PCR), Western blotting and flow-cytometry tests.

### Real-time quantitative PCR

Total RNA was extracted from tissues and cells with Trizol reagent (TaKaRa, Otsu, Japan) according to the specifications of the manufacturer. NanoDrop 2000 (Thermo, USA) was used to measure RNA concentration and reverse transcription kits (TakaRa) were used to obtain cDNA from RNA. SG (SYBR green kits) and a Bio-Rad iQ™5 system (Bio-Rad, USA) were used for real-time quantitative PCR following the specifications of the manufacturer. Mouse GAPDH was regarded as a housekeeping gene. We used a specific procedure and primer for the miRNA experiment. Mouse U6 was regarded as a housekeeping gene for miRNA. The levels of miRNAs were quantified by qRT-PCR using Bulge-Loop™ miRNA qRT-PCR Primer Sets (RiboBio, Guangzhou, China) and analysis performed on a Bio-Rad iQ™5 system (Bio-Rad). The primers for miR-129-5p and U6 small nuclear RNA were obtained from RiboBio Company (Guangzhou, China) and the sequences are covered by a patent. The primer sequences used for RT-qPCR analyses are listed in Table 1.

**Table 1.** Primer sequences used for real-time qPCR.

Name	Forward	Reverse
Cyclin E	CAGAGCAGCGAGCAGGAGC	GCAGCTGCTTCCACACCACT
Cyclin D	TAGGCCCTCAGCCTCACTC	CCACCCCTGGGATAAAGCAC
Cyclin B	AACTTCAGCCTGGGTCG	CAGGGAGTCTTCACTGTAGGA
P27	TGGGTCTCAGGCAAACTCT	TGGGTCTCAGGCAAACTCT
G3BP1	AACTCTTCCTATGCCCACGG	GTAGCCCCATCACTTGGACC
CDK6	TGGATAAAGTTCCAGAGCCCG	TTCTGCGGTTTCAGATCACGA
GAPDH	TGCTGAGTATGTCGTGGAGTCT	ATGCATTGCTGACAATCTTGAG

### Western blotting analysis

The 3T3-L1 cells were harvested and washed twice with PBS, then radio immunoprecipitation assay (RIPA) (Beyotime, China) buffer add with protease inhibitor mix (Pierce, USA) were added to 3T3-L1 cells under low temperature (4°C). Then, we used a cell scraper to separate the cell lysate from the culture plate as much as possible, which was then subjected to 4°C centrifugation (13,000*g*) for 10 min. Next, one-fourth volume of 5× loading buffer was added to the supernate and the mixture boiled for 10 min in water. A volume of 15–20 µg of protein samples was used for electrophoresis on 10% sodium dodecyl sulfate-polyacrylamide gel and then the proteins were transferred to a polyvinylidene difluoride (PVDF) membrane (Millipore, USA). The membrane was blocked at 4°C for 2 h with 5% milk, incubated with antibodies (1:500) against Cyclin D (Santa Cruz, USA), p27 (Santa Cruz), p38 (Cell Signaling, USA), phosphorylation of p38 (Cell Signaling), G3BP1 (Bosterbio, USA) and β-tubulin (Sungene Biotech, Tianjin, China) overnight at 4°C, followed by incubation with secondary antibody for 1.5–2 h at 4°C. Chemiluminescence reagents (Millipore, USA) were used to visualize the protein bands and the Image Lab software was used to quantify them.

### CCK-8 detection

3T3-L1 preadipocytes were cultured and subcultivated in 96-well plates at a density of 4000 cells per well. After treatment with miR-129-5p mimic or NC for 48 h, the cells were incubated in a medium containing 10 μL CCK-8 reagent for 4 h and the absorbance was measured.

### 5-Ethynyl-2′-deoxyuridine (EdU) imaging assay

A Cell-Light Edu Apollo 567 In Vitro Kit (RiboBio, Guangzhou, China) was used to detect the amount of cells present in the DNA synthesis phase. 3T3-L1 preadipocytes were subcultivated in 96-well plates at a density of 4000 cells per well and treated with EdU Reagent A (RiboBio, Guangzhou, China) for 4 h. Hoechst stain was used for cell nuclei staining. Images were captured and analyzed with a Nikon TE2000 microscope (Nikon, Tokyo, Japan).

### Flow cytometry

3T3 preadipocytes were cultured in 6-well plates at a density of 6×10^5^ cells per well and transfected with miR-129-5p mimic or NC 24 h later by using Roche Transfection Reagent. 3T3-L1 preadipocytes were washed three times with PBS and harvested by trypsin digestion 48 h after transfection. Harvested preadipocytes were fixed in 70% cold alcohol overnight at −20°C and treated with 1 mg/mL RNase A at 37°C for 45 min, then 50 mg/mL propidium iodide (PI) was used for staining for 30 min. Finally, the cell cycle of these cells was analyzed by a flow-cytometry instrument (Becton Dickinson, Franklin Lakes, NJ).

### Bioinformatics analysis

Target genes of miR-129-5p were predicted by TargetScan 7.1 (http://www.targetscan.org/vert_71) and RNAhybrid (https://bibiserv.cebitec.uni-bielefeld.de/rnahybrid).

### Luciferase reporter assays

We obtained the 3′UTR of G3BP1 which includes the miR-129-5p target sites from the cDNA of mouse WAT by PCR. The primers of the PCR were added to protective base and restriction sites of Xho I and Not I (TaKaRa). All constructs were confirmed by sequencing. PsiCHECK-2 Vectors (Promega, USA), which were sited downstream of Renilla gene, were linked with 3′UTR of G3BP1. The vectors were confirmed by restriction enzyme digestion and DNA sequencing. We also constructed G3BP1 3′UTR mutation luciferase vectors by replacing CAAA to TGGG at miR-129-5p binding sites. HEK293T cells were seeded in 48-well plates and transfected with miR-129-5p mimic or NC along with the above vectors 24 h later by using Roche Transfection Reagent. After treatment for 48 h, the DualGlo Luciferase Assay System (Promega, USA) was used to measure and analyze the relative luciferase activity of firefly and Renilla.

### Statistical analysis

GraphPad Prism 6 was used to make graphs. Quantitative results were represented as mean ± SEM. Statistical differences were assessed by Student’s multiple *t*-tests between different groups. *P* < 0.05 is considered to be of a significant difference. *P* < 0.01 is denoted as an extremely significant difference.

## Results

### Expression patterns and conservation analysis of miR-129-5p

**Figure 1.**
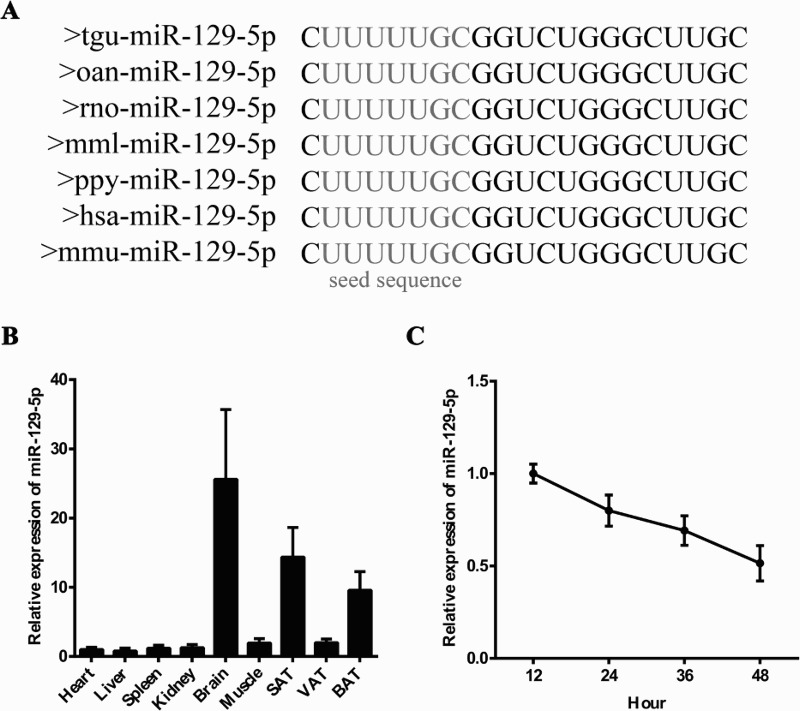
MiR-129-5p tissue distribution and expression pattern during 3T3-L1 preadipocyte proliferation. (A) Mature miR-129-5p sequence was highly conserved between different species. (B and C) The mRNA expression level of miR-129-5p in nine different mouse tissues (B) and during 3T3-L1 preadipocyte proliferation (C).

The expression profiles and conservation of miR-129-5p should be cleared in order to explore the function of miR-129-5p. The sequence of mature miR-129-5p was highly conserved in various species such as humans, mouse and rat (Figure 1(A)). To examine the expression patterns of miR-129-5p in mouse tissues, nine different tissues were harvested from 10-week-old C57BL mouse. Results analyzed by RT-qPCR suggested that miR-129-5p was widely expressed in mouse tissues and relatively highly expressed in brain, SAT and BAT (Figure 1(B)). Then, we harvested 3T3-L1 cells at 12, 24, 36 and 48 h after seeding to detect the miR-129-5p expression pattern during preadipocyte proliferation. It was shown that the expression level of miR-129-5p decreased during the proliferation process (Figure 1(C)). Since its high expression in SAT and decreased tendency during 3T3-L1 cells proliferation, we speculate that miR-129-5p might play a crucial part in this process.

### The enhanced miR-129-5p expression level inhibited DNA synthesis

**Figure 2.**
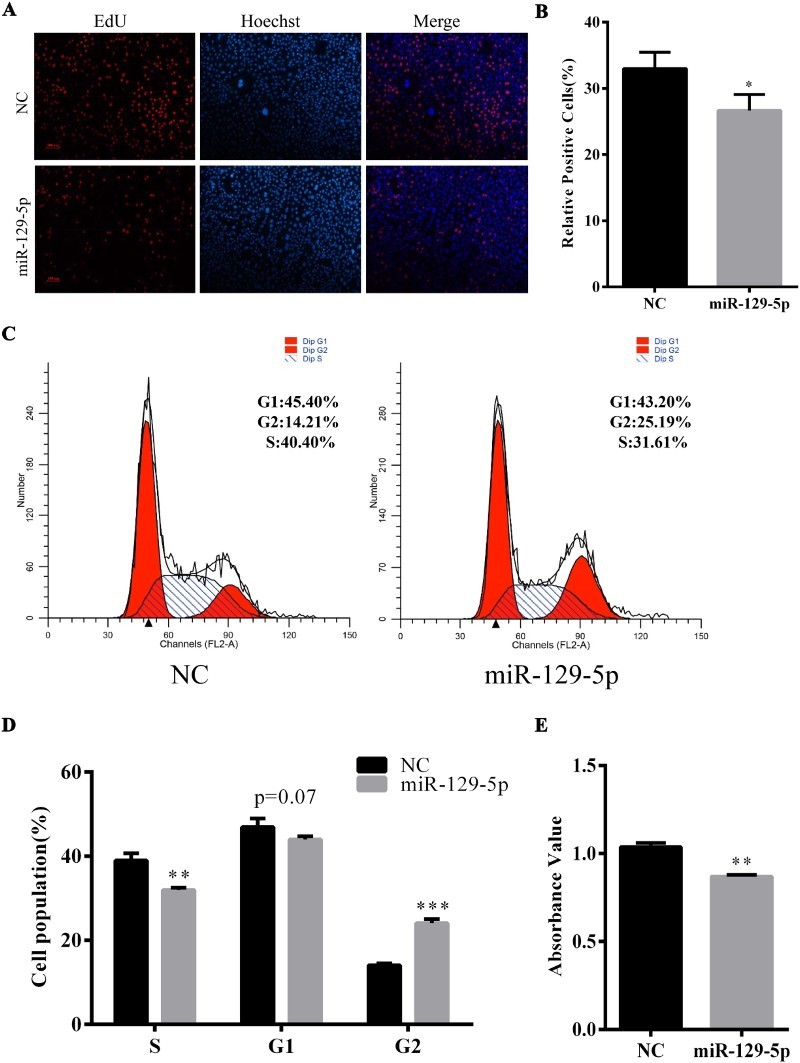
Overexpression of miR-129-5p significantly inhibited the proliferation of 3T3-L1 preadipocytes. (A) EdU incorporation assay of 3T3-L1 cells at 48 h after transfection. EdU staining (red). Cell nuclei were stained with Hoechst 33342 (blue). (B) The percentage of EdU-positive preadipocytes was analyzed. (C) Cell-cycle analysis of 3T3-L1 preadipocytes by flow cytometry at 48 h after transfection. (D) The statistical results of flow cytometry. (E) Cell vitality was tested by using Cell Counting Kit-8. Results are shown as means ± SEM. *n* = 3. * indicates *P* < 0.05, ** indicates *P* < 0.01, *** indicates *P* < 0.001.

To probe the possible function of miR-129-5p during preadipocyte proliferation, miR-129-5p mimic or NC was transfected into 3T3-L1 preadipocytes. Results showed that the proportion of EdU-labeled cells was significantly decreased in the miR-129-5p mimic-treated group compared with that in the NC group (Figure 2(A,B)). Also, the results of flow cytometry manifested that the enhanced miR-129-5p level decreased (*P* < 0.01) the proportion of cells in the S-phase and increased the G2-phase proportion (*P* < 0.001) (Figure 2(C,D)). Consistently, CCK-8 results showed a significant reduction in viable cell number when miR-129-5p was overexpressed (Figure 2(E)). Collectively, all the above data demonstrated that miR-129-5p inhibited DNA synthesis during 3T3-L1 cells proliferation.

### MiR-129-5p impaired the expression level of cell-cycle genes

**Figure 3.**
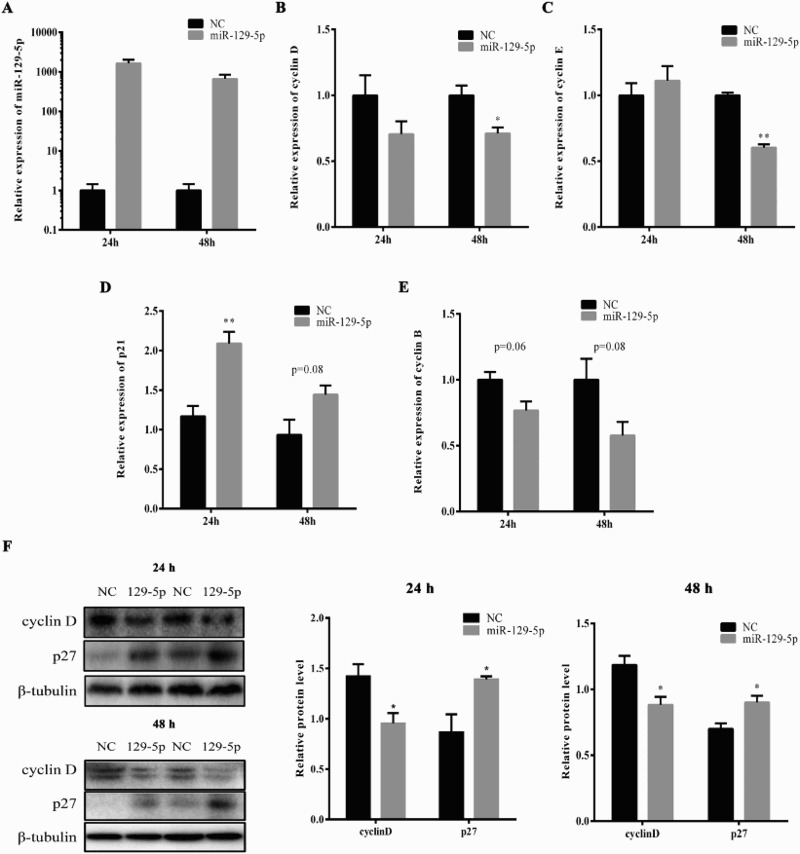
The enhanced miR-129-5p level in 3T3-L1 cells impaired the expression of cell-cycle genes. 3T3-L1 preadipocytes were transfected with miR-129-5p mimic or NC when reaching 40% confluence. (A) The overexpression efficiency of miR-129-5p was tested at 24 and 48 h after transfection, respectively. (B–E) The mRNA expression of cyclin D (B), cyclin E (C), p21(D) and cyclin B (E) was detected at 24 and 48 h after transfection by real-time qPCR. (F) The protein levels of cell-cycle genes were detected at 24 and 48 h after transfection by Western blotting. β-Tubulin was used as a reference gene. Results are shown as means ± SEM. *n* = 3. * indicates *P* < 0.05, ** indicates *P* < 0.01.

In order to further elucidate the miR-129-5p function during 3T3 preadipocyte proliferation, miR-129-5p mimic or NC was transfected into 3T3-L1 preadipocytes and the expression level of proliferation-related genes was detected 24 and 48 h later. The overexpression efficiency of miR-129-5p mimic was examined at 24 and 48 h. MiR-129-5p was 1600-fold overexpressed at 24 h and also 700-fold overexpressed at 48 h after transfection by miR-129-5p mimic (Figure 3(A)). The mRNA expression of cyclin D and cyclin E was significantly suppressed by miR-129-5p mimic at 48 h (Figure 3(B,C)), while p21 was significantly up-regulated at 24 h (Figure 3(D)). The mRNA level of cyclin B tends to be decreased at 24 and 48 h, but the results were not significant (*p* = 0.06 at 24 h and *p* = 0.08 at 48 h) (Figure 3(E)). Consistent with these results, cyclin D protein was significantly inhibited and the protein level of p27 was up-regulated in miR-129-5p mimic-treated cells as compared with NC at both 24 and 48 h (Figure 3(F)). These results also supported the suppression role of miR-129-5p during 3T3-L1 preadipocyte proliferation.

### MiR-129-5p targeted the 3′UTR of G3BP1 and activated the p38 signaling pathway

**Figure 4.**
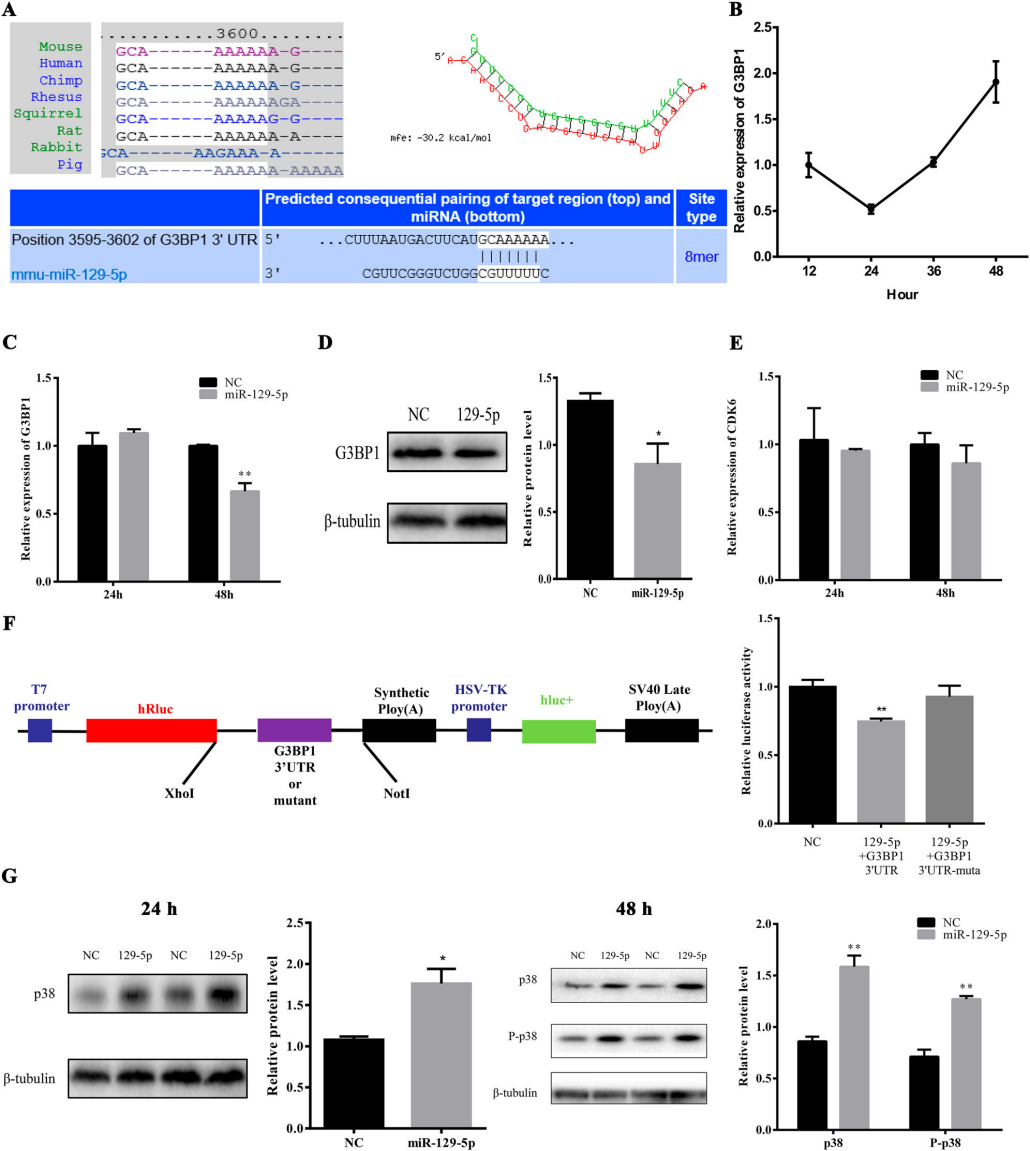
MiR-129-5p targeted G3BP1 3′UTR and activated the p38 signaling pathway. (**A**) Seed sequence of miR-129-5p targeted the 3′UTR of G3BP1. (B) Expression level of G3BP1 during 3T3-L1 preadipocyte proliferation. (C and D) The mRNA (C) and protein (D) expression of G3BP1 was tested at 48 h after transfection. (E) The expression level of CDK6 responded to miR-129-5p mimic. (F) The psiCHECK2-G3BP1-3′UTR or psiCHECK2-G3BP1-3′UTR mutation plasmid was co-transfected with 50 nM miR-129-5p mimic or NC into HEK293T cells. The cells were harvested and luciferase reporter assays were carried out at 48 h. (G) The protein level or the phosphorylation level of p38 was detected at 24 and 48 h after transfection by Western blotting. Results are shown as means ± SEM. *n* = 3. * indicates *P* < 0.05, ** indicates *P* < 0.01.

MiRNAs always regulate the biological process by targeting the 3′UTR, 5′UTR or coding sequences of mRNAs and inhibiting the expression level of these. We predicted that mouse G3BP1 was targeted by miR-129-5p and the predicted targeting site was conserved between different species (Figure 4(A)). Interestingly, the expression of G3BP1 during 3T3-L1 preadipocyte proliferation declined in the early phase and increased significantly in the late phase which was reversed to miR-129-5p (Figure 4(B)). Then, mRNA expression of G3BP1 was detected in 3T3-L1 cells after transfection with miR-129-5p mimic or NC. The enhanced miR-129-5p expression dramatically suppressed the mRNA expression level of G3BP1 (Figure 4(C)). Consistent with this, the protein expression of G3BP1 was also inhibited by miR-129-5p mimic (Figure 4(D)). However, the expression of CDK6, which has been proved to be a miR-129-5p targeting gene in humans (Wu et al. 2010), did not show any change between miR-129-5p mimic- and NC-transfected cells (Figure 4(E)).

Luciferase reporter vectors containing wild-type and mutated G3BP1 3′UTRs were constructed to further clear the targeting interaction between miR-129-5p and G3BP1. Then, the luciferase reporter vectors were transfected into HEK293T cells with miR-129-5p mimic or NC. As we expected, the relative luciferase activity of G3BP1 3′UTR vectors was decreased about 25% (*P* < 0.01) when they responded to enhanced miR-129-5p level. However, this change was not observed in the mutated reporter vector (Figure 4(F)). Therefore, G3BP1 was directly targeted by miR-129-5p. Previous studies have revealed that G3BP1 increased the cell proliferation rate by suppressing PMP22 mRNA expression in MCF-7 cells (**Winslow et al. 2013**). However, in osteosarcoma cells, PMP22 was proved to suppress cell proliferation by up-regulating the phosphorylation level of p38 (Liu & Chen 2015). To demonstrate whether miR-129-5p also inhibited 3T3-L1 cells proliferation via the p38 signaling pathway, the protein level or phosphorylation level of p38 was detected after treatment with miR-129-5p mimic for 24 and 48 h by Western blotting. As expected, the protein level and the phosphorylation level of p38 were significantly up-regulated in miR-129-5p mimic-transfected group (Figure 4(G)). Together, our findings identified miR-129-5p as a key repressor of preadipocyte proliferation by targeting G3BP1 and activating the p38 signaling pathway.

## Discussion

The increase in fat mass and following disorders of metabolism were caused by the preadipocyte proliferation and differentiation into mature adipocytes (Spiegelman & Flier 1996). A miRNA microarray analysis by Central South University revealed that miR-129-5p produces a 43-fold change in insulin-resistant adipocytes compared with that in normal adipocytes (Ling et al. 2009), suggesting that miR-129-5p may impair adipocytes number. MiR-129-5p has been reported to regulate cell proliferation in human tumor cells (Wu et al. 2010) and it was highly conserved among different species such as humans and mouse. Thus, we wonder whether miR-129-5p plays a crucial role in preadipocyte proliferation. In our research, we observed that the mRNA level of miR-129-5p decreased during 3T3-L1 preadipocyte proliferation. Also, miR-129-5p impaired DNA synthesis and the expression of proliferation-related genes to suppress 3T3-L1 preadipocyte proliferation. These data indicated that miR-129-5p is a negative regulatory factor of 3T3-L1 preadipocyte proliferation.

MiR-129-5p has been regarded as an important tumor suppressor. Researches have shown that miR-129-5p could regulate cell proliferation in thyroid cancer (Brest et al. 2011), lung adenocarcinoma (Wu et al. 2010), hepatocellular carcinoma (Liu et al. 2012a) and gastric cancer cell lines (Yu et al. 2013). Considering the differences among tissues and cell lines, miR-129-5p would regulate cell proliferation through different molecular mechanisms. Wu et al. showed that miR-129-5p caused G1-phase arrest by targeting CDK6 in human lung adenocarcinoma cells (Wu et al. 2010). While in our research, flow-cytometry results indicated that miR-129-5p impaired G2 but not G1 phase. CDK6 was found to be a crucial regulator of G1/S-phase transition by targeting the retinoblastoma protein (Rb) (Kozar & Sicinski 2005). We also detected the mRNA level of CDK6 at 48 h after transfection with miR-129-5p mimic and found that the CDK6 expression level did not change when it responded to an enhanced miR-129-5p level, as shown in Figure 4(D). Interestingly, our research revealed that miR-129-5p activated p38 signal transduction pathways, which have been reported to inhibit G2/M transition in cardiomyocytes (Engel et al. 2005). And, this may explain the G2-phase arrest phenomenon in our research.

G3BPs, Ras-GTPase-activating protein SH3 domain-binding proteins, are composed of three homologous proteins G3BP1, G3BP2a and G3BP2b (Kennedy et al. 2002). A previous study has demonstrated that G3BP1 depletion decreased the cell number and the cell proliferation rate by inhibiting PMP22 at the transcriptional level. The knockdown of G3BP1 facilitated PMP22 mRNA expression, but did not influence the mRNA stability of PMP22 (**Winslow et al. 2013**). In osteosarcoma cells, PMP22 was proved to suppress cell proliferation, and Western blot results showed that the phosphorylation level of p38 was up-regulated by enhanced PMP22 expression (Liu & Chen 2015). In our research, we discovered that the G3BP1 expression level showed an opposite tendency to miR-129-5p during 3T3-L1 cells proliferation, and both mRNA and protein levels of G3BP1 were decreased in miR-129-5p mimic-transfected cells compared with that in the NC group. The 3′UTR luciferase reporter assays further ensured that G3BP1 was directly targeted by miR-129-5p in 3T3-L1 preadipocytes. The p38 signaling pathways played a crucial part in the cell proliferation regulation of various cells by sharing the substrate and cross-cascade interaction (Zhang & Liu 2002). A lot of microRNAs have been reported to impair cell proliferation through the p38 signaling pathway. The down-regulation of the miR-1/133 expression level mediated by p38 could promote myoblast proliferation in muscle regeneration (Zhang et al. 2012). MiR-139 regulated human colorectal carcinoma cell proliferation by targeting RAP1B accompanied by a large change in the phosphorylation level of p38 (Guo et al. 2012). The phosphorylation level of p38 was up-regulated in miR-129-5p mimic-treated 3T3-L1 preadipocytes, which showed that miR-129-5p may function through the p38 signaling pathway. In a word, our data suggested that miR-129-5p suppressed 3T3-L1 preadipocyte proliferation by targeting G3BP1 and activating the p38 signaling pathway.

**Figure 5.**
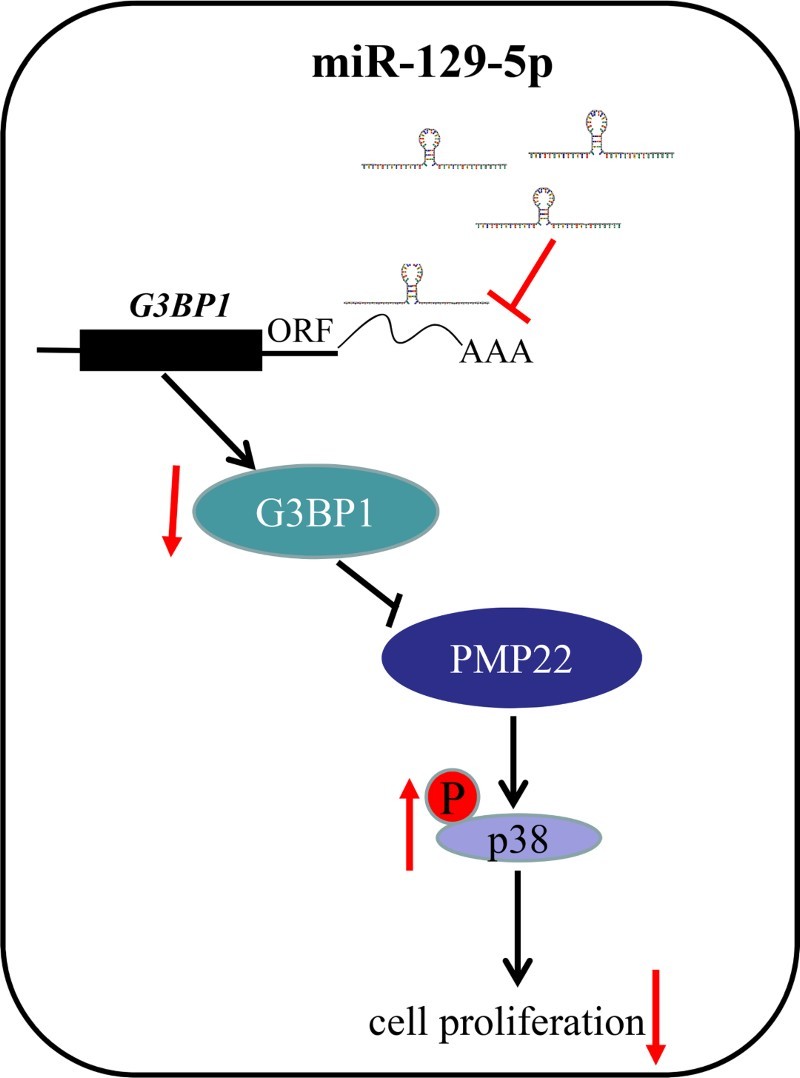
Schematic diagram of the negative regulation role of miR-129-5p in 3T3-L1 preadipocyte proliferation. During 3T3-L1 preadipocyte proliferation, miR-129-5p targeted G3BP1 3′UTR to inhibit its expression, then the decreased G3BP1 might regulate the expression of cell-cycle genes by increasing the phosphorylation level of p38. The red arrows indicate our work and black arrows indicate the existing researches.

In summary, as shown in Figure 5, overexpression of miR-129-5p inhibited the proportion of cells in the S-phase and impaired the expression of cell-cycle genes during 3T3-L1 preadipocyte proliferation. Our findings also indicated that miR-129-5p suppressed 3T3-L1 cells proliferation by targeting G3BP1 and up-regulating the expression of p38 and the phosphorylation level of p38. Further research will reveal any other target genes and signaling pathway involved in the negative regulation role of miR-129-5p during 3T3-L1 preadipocyte proliferation.
